# HIV-positive status disclosure to a sexual partner and associated factors among HIV-positive pregnant women attending antenatal care in Dire Dawa, Ethiopia: A cross-sectional study

**DOI:** 10.1371/journal.pone.0250637

**Published:** 2021-04-27

**Authors:** Mulusew Ambissa, Endalew Gemechu Sendo, Yeshi Assefa, Alemu Guta

**Affiliations:** 1 Black Lion Specialized Hospital, College of Health Sciences, Addis Ababa University, Addis Ababa, Ethiopia; 2 School of Nursing & Midwifery, College of Health Sciences, Addis Ababa University, Addis Ababa, Ethiopia; 3 Department of Midwifery, College of Medicine and Health Sciences, Dire Dawa University, Dire Dawa, Ethiopia; 1. IRCCS Neuromed 2. Doctors with Africa CUAMM, ITALY

## Abstract

**Introduction:**

Pregnant women who disclose their HIV-positive status to their sexual partners have played an important role in reducing the risk of HIV/AIDS transmission to the baby during the antepartum, intrapartum, and postnatal periods. Studies are limited in the current study area in a similar arena. Therefore, this study aimed to assess the proportion of HIV-positive status disclosure and its associated factors among pregnant women.

**Methods:**

A facility-based cross-sectional study was conducted among 156 HIV-positive pregnant women in Dire Dawa administrative from March 12^th^ to May 10^th^, 2020. Data were generated using a pretested structured questionnaire through face-to-face interviews. Binary logistic regression analysis was employed to identify the predictor variables associated with the disclosure of HIV-positive status among pregnant women to their sexual partners. Finally, the adjusted odds ratio with 95% confidence intervals at P-value< 0.05 was considered statistically significant.

**Results:**

Of the total, 135 (86.5%) of HIV-positive pregnant women disclosed their HIV status to their sexual partner. Christian followers (both Orthodox and Protestant) [AOR = 8.8, 95% CI: 2.3. 34] more likely to disclose HIV status to their sexual partner than those Muslims. Those participants who started practicing safer sex [AOR = 17.6, 95% CI: 4–77] and those women who had a smooth relationship before the HIV disclosure were [AOR = 14.7, 95% CI: 3–68.6] more likely to disclose HIV status to their sexual partner than their counterparts, respectively.

**Conclusions:**

The proportion of HIV serostatus disclosure by HIV-positive pregnant women attending antenatal care services to their sexual partners was encouraging. However, this does not mean that there is no need for further awareness and intervention. Hence, interventions to boost and support women in safely disclosing their HIV-positive status are needed.

## Introduction

Globally, in 2019, females accounted for 48% of all new HIV infections, and in sub-Saharan Africa, they accounted for 59% of all new HIV infections. Globally, 1.8 million and in Ethiopia, 44,000 children are living with HIV in the same year [1]. In Ethiopia, about 670,000 people were living with HIV in 2019, and women older than 15 years accounted for 390,000 (58.2%) of the total [2].

HIV-positive status disclosure to a sexual partner leads to safer sexual practices, prevention of re-infection, social and emotional, financial, and encourages partners to make informed reproductive health choices [3, 4]. It is also a crucial goal in HIV testing and counseling, and it increases the involvement of a woman partner in the prevention of mother-to-child transmission services [5–10]. However, disclosure remains a challenge for many pregnant women [9].

Also, disclosure enables HIV-positive pregnant women to understand HIV infection and make sense of their disease-related experiences and take part in healthcare decision-making for good or improved adherence to antiretroviral therapy [3]. It leads to positive health outcomes for both the patient and partner. It improved care-seeking behavior like condom use, partner testing that reduces the risk of HIV transmission, and social support, emotional well-being, improve long-term HIV care engagement among pregnant women, and an improved outlook for the future [11–14].

Despite this positive outcome, disclosure is a complex process that requires caution to prevent the occurrence of a bad outcome. It is a time of conflict with a partner, abuse, and withdrawal of finances [12, 15–18]. While HIV care engagement is vital during the antenatal period to PMTCT, it is a time of barriers to disclosure that happened [19]. To overcome these barriers, couple testing, advanced counseling, and overcoming the problem of stigma are the strategies for enhancing disclosure and prevention of HIV transmission [17].

Despite HIV-positive status, nondisclosure is the major challenge for HIV/AIDS care, a few studies have stated these issues in our country [20]. In 2020, the Global HIV Prevention Coalition has launched a program in 2017 to decrease new HIV infections by 75% [21]. Similarly, Ethiopia’s developed National HIV Prevention Road Map is aligned with the national HIV/AIDS strategic plan 2015–2020 [22]. National HIV strategies implemented specific interventions focused on the PMTCT of HIV. It includes HIV testing for all mothers during ANC and counseling for those who test positive emphasis on ART and its adherence and also to encourage them to tell the HIV status to their sexual partners.

Around the world, mother to child transmission is a significant cause of HIV-infections among children, especially in developing countries. Around 60% of HIV infections occur among women in most parts of Africa, and the rate is as high as 40% among antenatal care attendees in Sub Sahara Africa [23]. In a study conducted in Cameroon, the risk of HIV transmission from mother to baby is 30–45% in the absence of any intervention [24]. With no intervention, about 35% of children born to HIV-positive women are at risk of being infected with the virus [25].

HIV-positive disclosure to sexual partners reduces the risk of HIV transmission to the baby during the antenatal, intrapartum, and postnatal periods [4]. One study revealed that disclosing one’s serostatus for their sexual partner was predicted to decrease the risk of HIV transmission by between 17.9% and 40.6% relative to non-disclosed [26]. Another study showed that serostatus disclosure would contribute to minimizing HIV transmission to the child by 41% [9]. The studies conducted in sub-Saharan Africa showed that HIV-positive status disclosure to sexual partners among HIV-positive pregnant women was lower than the general population of people living with HIV [27].

Moreover, the studies were limited in the current study area. Therefore, this study aimed to determine the prevalence of HIV-positive status disclosure to sexual partners and its associated factors among HIV-positive pregnant women in Dire Dawa.

## Methods and materials

### Study setting and period

We conducted the study from 12^th^ of March to 10^th^ May 2020 in Dire Dawa. Dire Dawa is 515 km away from the center, Addis Ababa to the East. Dire Dawa is one of two chartered cities in Ethiopia with the other Addis Ababa. Dire Dawa had about 506,936 total population consists of 248,298 males and 258,638 females. About 162,220 (32%) rural and 344,716 urban residents with an average household size of 4.5, and 2.5 a population growth rate. Dire Dawa administrative had 38 rural and 9 urban Kebeles.

Dire Dawa Administration has two public hospitals, 15 health centers, and 34 health posts. Of these, ten public health facilities (two hospitals and eight health centers) are providing PMTCT services. According to Central Statistical Agency (CSA) and Inner city fund (ICF), in 2018, the Dire Dawa administration is the 3^rd^ HIV prevalent region with the rate of 2.5% next to Gambella and Addis Ababa in Ethiopia. EDHS 2011 showed that 4.3% of reproductive-aged (15–49) women are living with HIV in Dire Dawa. We included all ten public health facilities (two hospitals and eight health centers) providing PMTCT in the Dire Dawa administrative. All ten public health facilities with a client flow of 5–18 HIV-positive pregnant women per month were included in the study. According to mini EDHS 2019, 83.8% of women are receiving antenatal care from a skilled provider in the Dire Dawa Administration.

### Study design and population

A facility-based cross-sectional was conducted among selected HIV-positive pregnant women attending antenatal care services in the public health facilities of Dire Dawa administrative.

### Sample size and sampling procedure

The sample size was obtained by single population proportion formula using the assumption, p-value from the previous study finding (89.7%) of pregnant women had disclosed HIV-positive to their sexual partner [10], 95% CI, and a margin of error-0.05. However, since the total number of HIV-positive pregnant women was 161, we included all of them in this study. Pregnant women who met the eligibility criteria were contacted through the midwives/nurses in charge of the maternal and child health units of the selected hospitals and health centers. They were informed about the study, including providing adequate information regarding the purpose, procedure, benefits, and risks of the study. Then, we used a consecutive sampling method to select the eligible participants. To be included in the study, the participants had to be women who had attended focused antenatal care (FANC) at least twice in the selected health facilities and tested positive for HIV infection, aged 15–49 years, could communicate well in Amharic (local working language) and had lived in the study area for at least 6 months. One hundred fifty-six (156) study participants who fulfilled the eligibility criteria were interviewed during their ANC visit.

### Data collection tools and procedure

Data were collected through face-to-face interviews using structured and pre-tested tools. We have adapted it based on different literature which, includes socio-demographic characteristics, disclosure status and health service-related practice, and psychosocial characteristics among HIV-positive pregnant women [10, 28, 29]. The questionnaire was designed in English and translated into the local Amharic language and then translated back to English. We used ten trained health professionals who work in the same health facilities for the collection of data, and one MSc midwife supervises the data collection process.

The quality of data was assured by pre-testing of data collection tools on 10% (17 pregnant women) in Hiwot Fana Referral specialized Hospital from Jan 31th- February 10th, 2020, and necessary corrections on the instrument were incorporated. Data collectors took training on the purposes, the significance of the study, the art of interviewing, and meanings of each question, and the ethical issue. Every day, the supervisor and investigators reviewed each questionnaire for completeness, and we offered the orientation to data collectors every morning.

### Measurement and definitions

**HIV serostatus disclosure** refers to the action of telling the status of the HIV-positive test result to a sexual partner and making it revealed and known by him. In this study, disclosure of HIV serostatus was measured by using the question "does your most recent sexual partner know your HIV status?" and answers are yes or no [10].

**Sexual partner** refers to a woman’s most recent male partner in marriage who is biologically responsible for her current pregnancy.

### Data processing and analysis

We checked data for consistency and entered Epi Data version 4.2 and then exported it to the Statistical Package for Social Sciences (SPSS) version 24.0 software for analysis. Descriptive statistics like frequency, proportion, mean, and standard deviation computed to describe study variables about the population. Both bivariate and multivariate Logistic regressions analyses were used to determine the effect of independent variables on the outcome variables. Variables found to have a P-value <0.2 in the bivariate logistic regression entered/exported into multivariate analysis to identify the predictor variables. Then, the adjusted odds ratio with 95% CI at P-value< 0.05 was considered a statistically significant association with the outcome variable.

### Ethical consideration

We obtained ethical clearance from the institutional review board of Addis Ababa University with approval protocol number: 051/20/SNM. Then, a support letter with Ethical clearance was offered to Dire Dawa administration Regional Health Bureaus, health facilities (hospital and health centers). Permission was obtained from each center’s administrative body. Participation of all respondents was voluntary and written informed consent was ensured before engaging in the study. Written consent was also obtained from parents and/or guardians of the minors included in the study. Measures were taken to assure the respect, norms, values, beliefs, culture, and freedom of each individual participating in the study. Information on the purpose and procedures of the study was explained; confidentiality was maintained by omitting their identifications such as names, and a great deal of care and information was assured verbally to all study participants. The interview was carried out in a private room.

## Result

### Sociodemographic characteristics of participants

A total of156 pregnant women were interviewed. The mean age of the participants was 30.69 (SD ±3.957) years. Of 156 respondents, 59 (37.8%) were housewives, and fifty-four (34.6%) of the participants attended primary school. More than half, 84 (53.8%) had established a sexual relationship for two years and below with their partner **(Table 1)**.

**Table 1:** Sociodemographic characteristics of HIV-positive pregnant women attending ANC service in public health facilities in Dire Dawa, Ethiopia, 2020, (n = 156).

Variables	Number	Percent (%)
**Age (years)**		
15–29	51	32.7
30 and above	105	67.3
**Ethnicity**		
Oromo	75	48.1
Amhara	57	36.5
Tigre	6	3.8
Gurage	9	5.8
Somali	6	3.8
Others	3	1.9
**Religion**		
Orthodox	63	40.4
Muslim	69	44.2
protestant	24	15.4
**Marital status**		
Married	101	64.7
single	17	10.9
Divorced	30	19.3
Widowed	8	5.1
**Educational level**		
No formal education	36	23.1
Primary (1–8)	54	34.6
Secondary and above	66	42.3
**Occupation**		
Government employee	15	9.6
Private employee	24	15.4
Housewife	59	37.8
Daily labor	27	17.3
Merchant	21	13.5
Commercial sex worker	3	1.9
Others	7	4.5
**Duration of relationship with partners (year)**		
Two years and below	84	53.8
Above two years	72	46.2

*Hadiya, **Student, Jobless.

### HIV-positive status disclosure to a sexual partner

According to the finding, 135 (86.5%) pregnant women shared their HIV-positive status with their sexual partners. Following disclosure, 72 (46.2%) of the male sexual partners were supportive of their partners. Regarding the time of disclosure, 81 (51.9%) disclosed within the 1^st^ month of diagnosis, and 87 (55.8%) of women known their HIV status during ANC. Nearly half 75 (48.1%) of the mothers had sex with a partner before disclosure, among those 48 (30%) of them used condoms. Over half, 90 (57.7%) of the study participants were given information about HIV before testing. Participants who disclose HIV status to others was 80 (51.9%) and started practicing safer sex was 133 (85.3%) (Table 2).

**Table 2:** Seropositive HIV status disclosure experience by HIV-positive pregnant women attendant ANC service in public health facilities in Dire Dawa, Ethiopia, 2020, (n = 156).

Variables	Number	Percent (%)
**Partner’s reaction to HIV status disclosure**		
Supportive	72	46.2
Worried about his own HIV status	39	25.0
Blamed me to infect him	6	3.8
Talked about divorcing me	9	5.8
Other	9	5.8
**Duration of time for disclosure since diagnosed**		
Less than 1month	81	51.9
1–2 month	33	21.2
3–4 months	15	9.6
More than 4 months	6	3.8
**Have you sex with your partner before disclosure**		
Yes	75	48.1
No	60	38.5
**If yes, did you use a condom**		
Yes	48	30.8
No	27	17.3
**Given any information about HIV before you tested**		
Yes	90	57.7
No	66	42.3
**Information that was given by the health care worker**		
General about HIV	33	21.2
HIV transmission only	27	17.3
HIV prevention only	21	13.5
Positive living	3	1.9
**Disclosure of HIV status to others**		
Yes	81	51.9
No	75	48.1
**Started practicing safer sex**		
Yes	133	85.3
No	23	14.7

### Reason for disclosure HIV status to a partner

**Fig 1:**
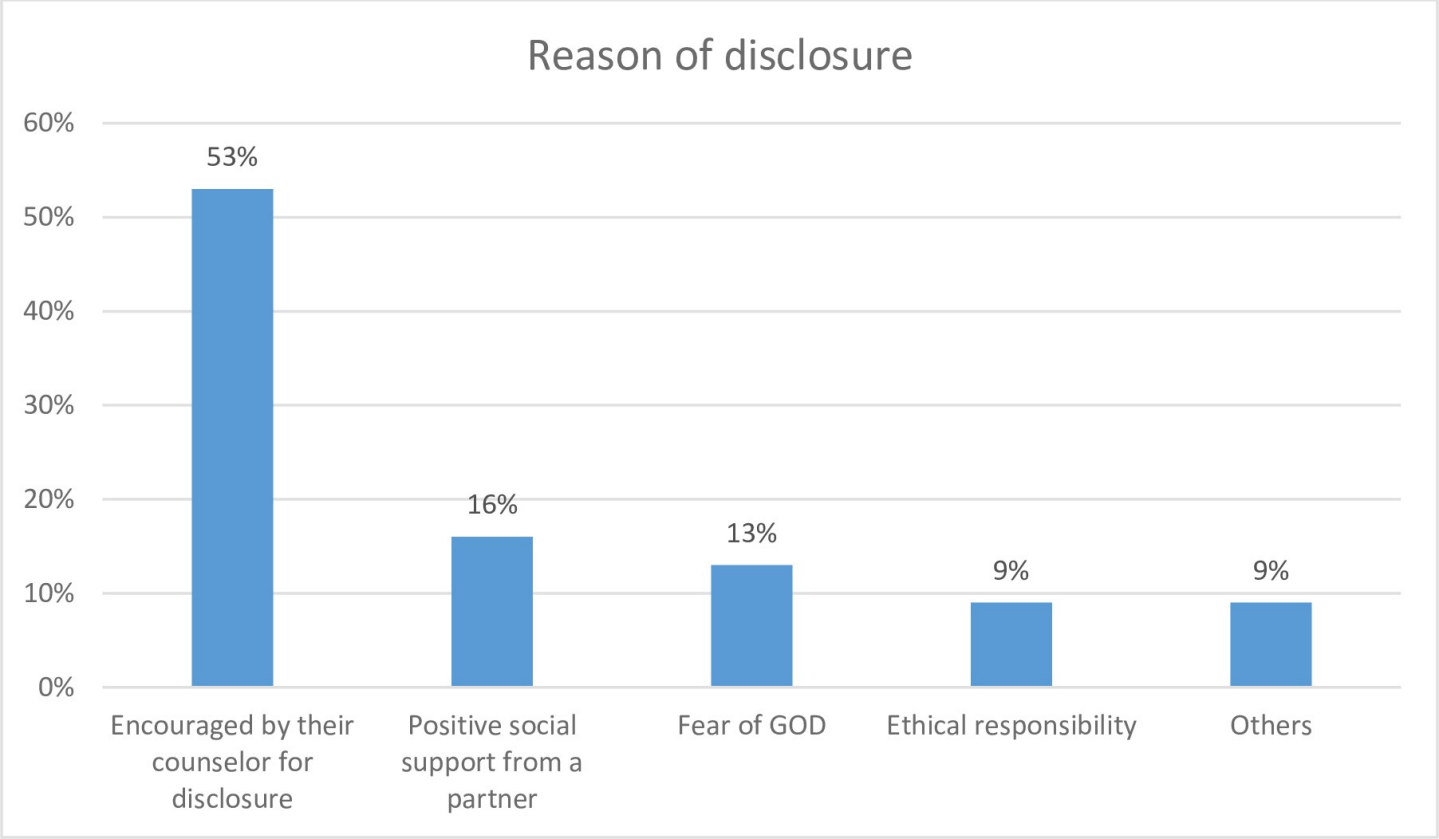
Participants’ reasons for disclosure of their HIV-positive status to a partner.

The reason for the disclosure, 83 (53%) of pregnant women were reported encouragement from their counselors about disclosure, followed by having positive social support from a partner **(Fig 1).**

### Psychosocial factors of HIV-positive pregnant women

Of the total, 72 (46.2%) of them had challenges of telling their HIV test result to their sexual partners; of these 30 (19.2%) of them reported fear of lack of support from their partner. Over half, 83 (53.2%) of participants have self-stigma, and 72 (46.2%) of the women have also shared their HIV-positive test results with their families. Regarding the relationship of participants with their partner before the HIV test, 138 (88.5%) of the participants have a smooth relationship (Table 3)

**Table 3:** Psychosocial factors among HIV-positive pregnant women attending ANC service in selected public health facilities in Dire Dawa, Ethiopia, 2020 (n = 156).

Variables	Number	Percent (%)
**Have you had challenges telling your sexual partner about your HIV + status?**		
Yes	72	46.2
No	84	53.8
**What are the challenges?**		
Fear of lack of support from their partner	30	19.2
Fear of the spread of information	12	7.7
Fear of abandonment	12	7.7
Fear of deterioration in the relationship with a partner	6	3.8
Fear of discrimination by family and community	6	3.8
Fear of violence	6	3.8
**Do the participants have self-stigma?**		
Yes	83	53.2
No	73	46.8
**Family know that you are HIV-positive**		
Yes	72	46.2
No	84	53.8
**If yes, who are they**		
Parents	39	25.0
Siblings	18	11.6
Children	6	3.8
All of them	9	5.8
**How they get to know about your HIV-positive status**		
I told them myself	36	23.1
They escorted me to get results	33	21.2
They got information from other people	3	1.9
**Relationship with your partner before the HIV test**		
Smooth relationship	138	88.5
With disagreement	18	11.5
**Relationship with your partner after disclosure of HIV test results**		
Smooth relationship	81	51.9
With disagreement	75	48.1

### Associated factors of HIV-positive status disclosure to sexual partners among HIV-infected pregnant women

In multivariate analysis, three factors are significantly associated, such as the religion of mothers, who started practicing safer sex, and relationships with a partner before the HIV test.

Those Christian followers “Orthodox and Protestants” were approximately 9 [AOR = 8.8, 95% CI, 2.3–34] times more likely to disclose HIV status to their sexual partner than those Muslim. Participants who started practicing safer sex were [AOR = 17.6, 95% CI, 4–77] times more likely to disclose HIV-positive to their sexual partner than those who did not practice. Another, those mothers who had a smooth relationship with their partner before the HIV test were 14.7 [AOR = 14.7 95% CI, 3–68.6] times more likely to tell HIV status to their partner than those mothers with a disagreement with their partners **(Table 4).**

**Table 4:** Factors associated with HIV-positive status disclosure to sexual partner among PMTCT attendees women in public health facilities in Dire Dawa, 2020, (n = 156).

Variables	Disclosure status (N = 156)		COR (95% CI)	AOR (95% CI)	P-value
	Yes (N = 135)	No (N = 21)			
**Marital Status (currently)**					
Married	91	10	2.27 (0.9–5.7)	1.045 (0.3–3.6)	0. 944
Not	44	11	1	1	
**Religion of women**					
Christian	81	6	3.75 (1.4–10.3)	8.8 (2.3–34)	0.002*
Muslim	54	15	1	1	
**Duration of relationship with partners (year)**					
0–2 year	69	15	1	1	
Above two years	66	6	2.4 (0.87–6.5)	3.78 (0.9–15.8)	0. 069
**HIV status of partner**					
Positive	63	6	1	1	
Negative	72	15	2.2 (0.8–5.9)	1.2(0.34–4.2)	0. 769
**Started practicing safer sex**					
Yes	123	12	7.7 (2.7–21.9)	17.6 (4–77)	0.000*
No	12	9	1	1	
**Relationship with a partner before the HIV test**					
Smooth relationship	123	12	7.6 (2.7–21.9)	14.7 (3–68.6)	0.001*
With disagreement	12	9	1	1	

*Statistically significant at p-value <0.05 in multivariate logistic regression analysis.

## Discussion

In this study, the overall prevalence of disclosure of HIV status to sexual partners among pregnant women LHIV+ was 86.5%. This was similar to that of studies from Nigeria (88%) [30], Uganda (85.4%) [16]. It is also comparable to the studies conducted in Gondar and Addis Ababa (2019), which was 89.7% and 80.6%, respectively [10, 31].

However, this finding is higher than the studies conducted in Uganda and Tanzania were 69% and 41%, respectively [9, 26]. Similarly, this higher than the studies conducted in a different place of Ethiopia in southwest Ethiopia, Addis Ababa (2013), and Mekelle which was 69%, 72.9%, and 57.4%, respectively [28, 29, 32]. This difference might be because of variations in the study population difference since the current study included specifically only HIV-positive pregnant women while the studies conducted in Uganda, Mekelle, and Southwest Ethiopia included all mothers, sampling size among others. The other reason may be the elapsed study period that leads there is encouraging disclosure by a health care professional, and improvement issues related to stigma, and there is access to PMTCT services with partner joint in the current study.

In this study, participants who started practicing safer sex were 17 times more likely to disclose HIV-positive to their sexual partner than those who did not practice. Studies do not support this finding. This might because starting or need to use a condom as unusual leads to questioning the sexual partner. This repeated question may lead to the disclosure of HIV test status.

Another, participants who had a smooth relationship with their partner before the HIV test were 14.7 times more likely to disclose HIV status to a sexual partner than those compared with have not. This finding agrees with many other studies conducted in Ethiopia [10, 28, 29]. The justification might be because the couple’s prior smooth relation leads to better communication, and also it avoids the secret between couples and avoids the fear of women to disclose.

## Conclusion

The proportion of HIV serostatus disclosure by HIV-positive pregnant women attending antenatal care services to their sexual partners was encouraging. However, this doesn’t mean that there will be no need for further awareness and intervention. Hence, interventions to boost and support women in safely disclosing their HIV + status are compulsory.

### Limitations

We should interpret the findings of this study considering its limitations. The result of this study depended on self-report of a sensitive topic disclosure of HIV status. As in all cross-sectional studies, we can infer association but not causation from our results. The findings of this study were limited to the study setting. The information obtained from women could also be subject to recall bias. Besides, not interviewing health workers and exclusion of HIV-positive pregnant women missing visits or whose follow-up visits not within the study period might affect the results of the study.

## Supporting information

S1 Questionnaire The data collection tool in the local language, Amharic. (DOCX)

S2 Questionnaire The data collection tool in English. (DOCX)

## Abbreviations and acronyms

AIDS: Acquired Immune Deficiency Syndrome
ANC: Antenatal Care
ART: Antiretroviral Therapy
CSA: Central Statistical Agency
ANC: Antenatal Care
FANC: Focused Antenatal Care
ICF: Inner city fund
HIV: Human Immunodeficiency Virus
PMTCT: Prevention of Mother to child transmission of HIV
