# Supplementary material for: HIV-positive status disclosure to a sexual partner and associated factors among HIV-positive pregnant women attending antenatal care in Dire Dawa, Ethiopia: A cross-sectional study
Source: PLoS One. 2021 Apr 27;16(4):e0250637. doi: 10.1371/journal.pone.0250637 (PMC8078815; doi:10.1371/journal.pone.0250637)
Supplement: S1 Questionnaire — (DOCX) [file pone.0250637.s001.docx]

**S1. Questionnaire (Amharic Version); መጠየቅ በአማረኛ**

**የምርምር ፕሮጀክቱ ርዕስ፡** የኤኤችአይቪ/ኤድስ በደማቸዉ ዉስጥ ያለባቸዉ እርጉዝ ሴቶች ለባለበታቸዉ ማሳወቃቸዉን እና የተያያዙ ጉዳዮችን በተመረጠ የመንግስት የጤና ተቋም በድሬዳዋ ከተማ ኢትዮጵያ በ2020።

**ክፍል ሀ: ሥነ-ህዝብና ማህበራዊ ጉዳዮች የተመለከተ መጠየቅ**

**መመረያ**፡ መልሱን ባዶ ቦታ ላይ ይፃፊ ወይም ምርጫ ከሆነ ይክበቡት፤

| **ተ.ቁ** | **ጥያቄ** | **ምላሽ** | **ይልፍ** |
| --- | --- | --- | --- |
| 001 | ዕድሜዎ ስንት ዓመት ነው? | …………………………(በዓመታት) |  |
| 002 | ብሔርዎ ምንድን ነው? | 1. ኦሮሞ 2. አማራ 3. ትግራዋይ 4. ጉራጋ 5. ሱማሌ 6. ሌላ ካለ ይጠቀስ...... |  |
| 003 | ሃይማኖትዎ ምንድነው? | 1. ኦርቶዶክስ 2. ሙስሊም 3. ፕሮቴንስታንት 4. ሌላ ካለ ይጠቀስ.................... |  |
| 004 | የጋብቻ ሁኔታዎ ምንድ ነው? | 1. ያገባ 2. ያላገባ 3. የተፋታ 4. ባሏ የሞተባት |  |
| 005 | የትምህርት ደረጃ ምንድ ነው? | 1. መደበኛ ትምህርት የለላት 2. የመጀመሪያ ደረጃ (1-8) 3. ሁለተኛ ደረጃ (9-12) እና ከዛ በላይ |  |
| 006 | ስራዎት ምንድን ነው? | 1. የመንግስት ተቀጣሪ 2. የግል ሰራተኛ 3. የቤት እመቤት 4. የቀን ሰራተኛ (ዕለታዊ የጉልበት ሥራ) 5. ነጋዴ 6. ሴተኛ አዳሪ 7. ሌላ ካለ ይጠቀስ... |  |
| 007 | ከአጋርሸ/ጓደኛ ጋር የነበረሽ ቆይታ ምን የህል ነዉ? | 1. 0-2 ዓመት  2. ከሁለት ዓመታት በላይ |  |

**ክፍል ለ: የኤችአይቪ ውጤትን ስለማሳወቅ እና ከጤና አገልግሎት ጋር የተያያዙ ጉዳዮች**

**መመረያ**፡ መልሱን ባዶ ቦታ ላይ ይፃፊ ወይም ምርጫ ከሆነ ይክበቡት፤

| **ተ.ቁ** | **ጥያቄ** | **ምላሽ** | **ይልፍ** |
| --- | --- | --- | --- |
| 008 | የኤችአይቪ / ኤድስ ውጤትዎን ለወሲባዊ ጓደኛዎ (ለባለቤትዎ) ነግረዋል? | 1. አዎ 2. አይደለም |  |
| 009 | ለጥያቄ 008 መልስዎ አዎ ከሆነ፤ ለአሁኑ አጋርሮ/ጓደኛዎ የኤች አይቪ ሁኔታ ለመግለጽ ምክንያቶች ምንድናቸው? | 1. የሥነምግባር ኃላፊነት  2. ከአማካሪ ማበረታቻ አግንቼ ነዉ  3. ከአጋሬ አዎንታዊ ማህበራዊ ድጋፍ  4. እግዚአብሔርን መፍራት  5. ሌላ (ይግለጹ)………………. |  |
| 010 | ለጥያቄ 008 መልስዎ **አዎ** ከሆነ፤ ስለ ኤች አይቪ ሁኔታ ለጓደኛሸ ስትገልጭ ምላሹ ምን ነበር? | 1. ደጋፊ 2. ስለራሱ የእራሱ ሁኔታ መጨነቅ 3. ለእሱ እንዳስተላልፍኩበት ተወቅሻለሁ 4. ስለፍቺ ማውራት ነገረኝ 5. ሌሎች (ይግለጹ)………………… 6. አያውቅም |  |
| 011 | ኤች አይ ቪ ተመርምረዉ ካወቁ በኋላ ለመግለጽ የጊዜ ቆይታ ምን ያህል ነበር? | 1. ከአንድ ወር በታች  2. 1-2 ወር  3. 3-4 ወራት  4. ከአራት ወር በላይ |  |
| 012 | እርስዎ ኤች አይ ቪ እንዳለብዎ ካወቁ በኋላ ጓደኛዎ ከማሳወቅዎ በፊት ከአሁኑ ባልደረባዎ ጋር የግብረ ሥጋ ግንኙነት ፈጽመው ያውቃሉ? | 1. አዎን  2. አልፈፀምኩም |  |
| 013 | ለጥያቄ 012 መልስዎ አዎ ከሆነ፤ ከእሱ ጋር ግንኙነት በሚያደርጉበት ጊዜ ኮንዶም ተጠቅመው ነበር? | 1.አዎን  2. አልተጠቀምንም |  |
| 014 | በኤች አይ ቪ ከመመርመሮ በፊት ስለ ኤች አይ ቪ የጤና መረጃ ሰጡት? | 1. አዎ  2) የለም |  |
| 015 | ለጥያቄ 014 መልስዎ አዎ ከሆነ፤ ምን መረጃ ተሰጥቶዎታል? | 1. ስለ ኤች.አይ.ቪ 2. የኤች.አይ.ቪ ስርጭት 3. የኤች.አይ.ቪ መከላከያ 4. አወንታዊ ኑሮ |  |
| 016 | የኤች አይ ቪ ሁኔታዎ ለሌሎች አሳውቀዋል? | 1. አዎን  2. አላሳወኩም |  |
| 017 | የኤች አይ ቪ ሁኔታዎ ካወቁ በኋላ ደህንነቱ የተጠበቀ ወሲባዊ ልምምድ ማድረግ ጀምረዋል? | 1. አዎን  2. አልጀመርንም |  |

**ክፍል : የግል ስነ ልቦናዊ ጉዳዮች**

**መመረያ**፡ መልሱን ባዶ ቦታ ላይ ይፃፊ ወይም ምርጫ ከሆነ ይክበቡት፤

| **ተ.ቁ** | **ጥያቄ** | **ምላሽ** | **ይልፍ** |
| --- | --- | --- | --- |
| 018 | ስለ ኤች አይ ቪ / ኤድስ ሁኔታዎ ለጓደኛዎ (ባለቤትዎ) ለመናገር አዳጋች ችግሮች አጋጥመውዎታል? | 1) አዎ  2 የለም |  |
| 019 | ለጥያቄ 018 መልስዎ አዎ ከሆነ፤ ስለ ኤች አይ ቪ / ኤድስ ሁኔታዎ ለወሲባዊ ጓደኛዎ ለመንገር ምን አይነት ተፈታታኝ ሁኔታዎች አጋጥመውዎታል? | 1) ለልጆቼ ክፍያዎች / ምግብ / ኪራይ / ድጋፍ / አለመኖር ፍርሃት  2) የመረጃ መስፋፋት ፍርሃት / ወሬ  3) የመተው ፍርሃት  4 ግንኙነታችን እንዳይሻክር በመስጋት  5) በቤተሰብ እና በማህበረሰቡ የመገለል ፍርሃት  6) ያለመተማመን  7) የጥቃት ፍርሃት  8) ሌሎች (ይግለጹ) |  |
| 020 | ተሳታፊው እራሱን የሚያገለል ነውን? | 1) አዎ  2) የለም |  |
| 021 | በቤተሰብዎ መካከል ኤች.አይ.ቪ ቫይረስ እንደለብዎ ያውቀዋል? | 1) አዎ  2) የለም |  |
| 022 | ለጥያቄ 021 መልስዎ አዎ ከሆነ፤ እነሱ ማን ናቸው? | 1) ወላጆች  2) እህትማማቾች  3) ልጆች  4) ሌሎች ይጥቀሳሉ....... |  |
| 023 | ስለ ኤች.አይ.ቪ ቫይረስዎ ያለበትን ሁኔታ እንዴት አወቁ? | 1) እኔ ራሴ ነግሬአቸዋለሁ  2) ውጤቶችን እንዳገኝ አስረዱኝ  3) ከሌሎች ሰዎች መረጃ አግኝተዋል  4) ሌሎች............ |  |
| 024 | ምርመራ ከማድረጎ በፊት ከባልደረባዎ ጋር ያለዎት ግንኙነት ምን ይመስል ነበር? | 1. ጥሩ ግንኙነት  2. አለመግባባት |  |
| 025 | ምርመራ አድርገዉ የኤች አይ ቪ ሁኔታዎ ለባልደረባዎ ከገለፁ በኋላ ከባልደረባዎ ጋር ያለዎት ግንኙነት ምን ይመስል ነበር? | 1. ጥሩ ግንኙነት  2. አለመግባባት |  |
