# Supplementary material for: HIV-positive status disclosure to a sexual partner and associated factors among HIV-positive pregnant women attending antenatal care in Dire Dawa, Ethiopia: A cross-sectional study
Source: PLoS One. 2021 Apr 27;16(4):e0250637. doi: 10.1371/journal.pone.0250637 (PMC8078815; doi:10.1371/journal.pone.0250637)
Supplement: S2 Questionnaire — (DOCX) [file pone.0250637.s002.docx]

**S2. Questionnaire English version**

**Topic: HIV-positive status disclosure to a sexual partner and associated factors among HIV-positive pregnant women attending antenatal care in Dire Dawa, Ethiopia: A cross-sectional study**

**Part I: Socio-demographic characteristics**

**Instructions:** Write your response on the space provided for open ended questions and encircle your response for close ended questions.

| **S. no** | **Question** | **Coding categories** | **Skip** |
| --- | --- | --- | --- |
| 001 | Age? | …………………… year |  |
| 002 | Ethnicity? | 1. Amhara 2. Oromo 3. Tigrae 4. Somale 5. Gurage 6. Other (specify)……….. |  |
| 003 | Religion? | 1. Orthodox 2. Muslim 3. Protestant 4. Other (specify)… |  |
| 004 | Marital status? | 1. Single 2. Married 3. Divorced 4. Widowed |  |
| 005 | Education status? | 1. No formal education 2. Elementary 1-8^th^ grade 3. Secondary and above |  |
| 006 | Occupation? | 1. Governmental employed 2. Private employee 3. Housewife 4. Daily labor 5. Merchant 6. Commercial sex worker 7. Other (specify) |  |
| 007 | Mother’s duration of relationship with their partners? | 1. 0-2 years 2. >2 years |  |

**Part II: HIV positive status disclosure and health service-related practice**

**Instructions:** Write your response on the space provided for open ended questions and encircle your response for close ended questions.

| **S. no** | **Question** | **Coding categories** | **Skip** |
| --- | --- | --- | --- |
| 008 | Have you disclosed your HIV results to your current sexual partner? | 1. Yes 2. No |  |
| 009 | If answer **yes** for Question-**008**, Why did you decide to disclose for your sexual partner about your HIV status? (Multiple answers possible)? | 1. Ethical responsibility 2. Encouragement from counselors 3. Positive social support from a partner 4. Fear of God 5. Other (Specify)……… |  |
| 010 | If answer **yes** for Question-**008**; What is your partner’s reaction to HIV status disclosure? | 1. Supportive 2. Worried about his own HIV status 3. Blamed me to infect him 4. Talked about divorcing me 5. Others (Specify)………..። 6. Don‘t know” |  |
| 011 | Duration of time for disclosure since you diagnosed as HIV positive? | 1. Less than 1month  2. 1- 2 month  3. 3- 4 months  4. More than 4 months |  |
| 012 | Have you ever had sex with your current partner before disclosure of your HIV + Status? | 1. Yes 2. No |  |
| 013 | If yes for question **012;** did you use condom while having sex with him?? | 1. Yes 2. No |  |
| 014 | Do you get information about HIV before you tested? Are you given any information about HIV by a health worker before you tested for HIV? | 1. Yes 2. No |  |
| 015 | If yes for question **014**; What information were you got? | 1. General about HIV 2. HIV transmission only 3. HIV prevention only 4. HIV positive living |  |
| 016 | Do you disclose your HIV-positive status to others? | 1. Yes 2. No |  |
| 017 | After knowing your HIV serostatus, have you started practicing safer sex? | 1. Yes 2. No |  |

**Part II: Psychosocial related factors**

**Instructions:** Write your response on the space provided for open ended questions and encircle your response for close ended questions.

| 018 | Have you had challenges telling your sexual partner about your HIV + status? | 1. Yes 2. No |  |
| --- | --- | --- | --- |
| 019 | If yes for question **018**; what challenges did you have? | 1. Fear of lack of support from my partner 2. Fear of spread of information/rumors 3. Fear of abandonment 4. Fear of deterioration in the relationship with partner 5. Fear of discrimination by family and community 6. Fear of being labelled promiscuous/unfaithful/infidelity 7. fear of violence 8. Others (specify) ……… |  |
| 020 | Do you have self-stigma? | 1. Yes 2. No |  |
| 021 | Does anyone in your family know that you are HIV-positive? | 1. Yes 2. No |  |
| 022 | If yes for question **021**; Who are they? | 1. Parents 2. Siblings 3. Children 4. All of them 5. Others specify......... |  |
| 023 | How did they get to know about your HIV positive status? | 1. I told them myself 2. They escorted me to get results 3. They got information from other people 4. Others specify......... |  |
| 024 | What is your relationship with your partner before HIV test? | 1. Smooth relationship 2. With disagreement |  |
| 025 | What is your relationship with your partner after disclosure of HIV test results? | 1. Smooth relationship 2. With disagreement |  |
